# A review of treatments targeting DNA-repair gene defects in metastatic castration resistant prostate cancer

**DOI:** 10.3389/fonc.2023.1150777

**Published:** 2023-03-14

**Authors:** Diana V. Maslov, Quinne Sember, Jason Cham, Munveer Bhangoo

**Affiliations:** ^1^ Department of Hematology/Oncology, Scripps Health System, San Diego, CA, United States; ^2^ Scripps Clinic/Green Hospital, Department of Internal Medicine, San Diego, CA, United States

**Keywords:** prostate cancer, DNA damage (DDR), metastatic, castration refractory prostate cancer (CRPC), target therapeutics

## Abstract

Prostate cancer is the most common cancer in men. About 6% of those diagnosed will develop metastatic disease. Unfortunately, metastatic prostate cancer is fatal. Prostate cancer can be castration sensitive or castration resistant. Many treatments have been shown to improve progression free survival and overall survival in metastatic castration resistant prostate cancer (mCRPC). In recent years, studies have been exploring targeting mutations in the DNA Damage Repair (DDR) response that may amplify oncogenes. In this paper, we aim to discuss DDR, new approved targeted therapies, and the most recent clinical trials in the setting of metastatic CRPC.

## Introduction

Prostate cancer is the most common cancer and second leading cause of cancer-related death in men (1–3). Androgen receptor signaling drives prostate cancer growth, and a mainstay of treatment for prostate cancer is androgen deprivation therapy (ADT) which interrupts the androgen receptor signaling cascade (4). However, some prostate cancers will progress despite appropriate suppression of testosterone as a result of androgen independent signaling (4). Despite the early clinical efficacy of ADT, 10-20% of patients will become resistant to ADT and develop castrate resistant prostate cancer (CRPC) within 5 years (5).

At present, there are multiple treatment options for metastatic CRPC. These treatments include second generation androgen receptor blockers, inhibition of androgen biosynthesis with an agent such as abiraterone (6), and taxane chemotherapy with docetaxel or cabazitaxel (7). There have been studies using mitoxantrone but multiple studies have shown increased toxicity with response rates between 9-20% (7–10). Radium 223 is also approved for use in castrate resistant prostate cancer with bone metastases (11). This is a radioactive isotope which is taken up in the bones and concentrates in areas of high osteoblastic activity (11, 12). In March, 2022, [177 Lu]Lu-PSMA-617 was approved for the treatment of prostate cancer. This works by targeting prostate-specific membrane antigen (PSMA) which is overexpressed in prostate tumor tissue. This is the first radiolabeled drug to be approved for CRPC (13). Despite the clinical benefit seen with these therapies, most patients will eventually progress. Therefore, there is an interest in identifying further targeted therapies.

Single nucleotide or oligonucleotide damage is repaired by either mismatch repair (MMR), base excision repair (BER) or nucleotide excision repair (NER) whereas larger DNA damage such as double stranded breaks (DSB) require homologous recombination (HR) or non-homologous end joining (NHEJ). Compared to NHEJ, HR results in an error free repair because it involves finding and synapsing with a homologous locus to prime repair (14). Specific DNA Damage Response (DDR) pathways are activated depending on the type of DNA insult and damage (15). If successful repair is not possible, programmed cell death is activated to maintain genomic integrity. Thus, mutations in DDR can lead to oncogene amplification and cancer progression (15). Here, we aim to discuss the clinical implications of DDR alterations and review the most recent clinical trials of targeted therapies for DDR mutations in the setting of CRPC.

## Homologous recombination repair (HRR) mutations

Homologous recombination repair (HRR) is a DNA repair process in which proteins seek homologous regions of chromosomes that may be damaged, copy the sequence from the undamaged strand, and resolve the structure (14). When there are mutations in these proteins, DNA is not repaired properly, which can lead to proliferation of cancer cells (14).

Mutations in BRCA1 and BRCA2 are known to be associated with an increased risk of prostate cancer in men (16). BRCA1 increases the risk of prostate cancer in patients less than 65 years old by 1.8 fold and BRCA2 by 4.5 times (16). Those with BRCA2 mutations tend to be diagnosed at a younger age, have higher Gleason grade tumors, and shorter survival (17). The PROREPAIR-B study found that those with BRCA2 mutation had a disease specific survival of about half than those without a DDR mutation (17.4 vs. 33.2 mos, p =.027) (18). Those with BRCA2 also had significantly lower survival than those with other non-BRCA2 mutations. Therefore, BRCA2 is an independent prognostic factor for survival in those with mCRPC. This is showing the importance of utilizing treatments in those with BRCA2 mutation. Polyadenosine diphosphate-ribose polymerase (PARP) inhibitors have been studied in multiple trials in patients with metastatic CRPC and BRCA1 and 2 mutations (19, 20).

### PARP inhibitors and HRR mutations

Many of the studies examining the use of PARP inhibitors in patients with BRCA 1 or 2 mutations also included patients with more rare mutations in homologous recombination repair genes. These genes include PALB2, ATM, CDK12, RAD51, and FANCA.

PALB2 is a gene which encodes a protein that links BRCA1 and 2 to form a surveillance complex (21). ATM works by halting the cell cycle when data replication is needed to repair. It is primarily involved in responding to DNA double-stranded breaks. In ATM-deficient cells, damaged DNA continues to replicate (22). A study by Neeb et al. (2021) detected ATM loss in 11% of patients with CRPC. ATM loss did increase genomic instability but did not seem to result in inferior clinical outcomes for patients (22). The PROREPAIR-B study found that in a patient population of 419 with mCRPC, 16% had an HHR mutation (BRCA1/BRCA2/ATM/[PALB2) (18).

The CDK12 gene is a tumor suppressor that encodes for cyclin-dependent kinase 12 (23). Cyclin dependent kinases (CDKs) regulate transcription by phosphorylating RNA polymerase which serves as a platform to recruit other transcriptional factors. Inhibition or loss of CDK12 has been shown to result in dysregulation of homologous recombination DNA repair genes such as BRCA1, FANCD2 and ATR, leading to genomic instability, tandem duplications, and gene fusions (23). CDK12 mutations occur in 4-11% of prostate cancers. The subset of prostate cancer with CDK12 mutations display more aggressive disease features such as a shorter time to metastasis and development of castration resistant disease (23).

RAD51 appears during S phase and is required to initiate stalled or broken DNA replication forks. It co-localizes with BRCA2 in mitotic cells (17). The interaction between BRCA2 and RAD51 is essential for error-free DNA synthesis to take place. RAD51 is known to be expressed more in aggressive prostate cancers, such as those with Gleason grades >7 (17).

Homozygous deletions of the FANCA gene occur in about 6% of prostate cancers, according to some studies (24). *In vitro* prostate cancer cells with FANCA deletions seem to have higher sensitivity to cisplatin and other DNA damaging substances compared to cells with wild-type FANCA (24). FANCA alterations also seem to sensitize cells to PARP inhibitors (24).

### Clinical trials

The TOPARP-B study was a phase II trial which evaluated two different doses of olaparib in patients with metastatic CRPC who had BRCA1 and 2 mutations as well as in patients with mutations in PALB2, ATM, and CDK12 (25). The primary endpoint of the TOPARP-B trial was composite response including radiological response as defined by RECIST 1.1 criteria, PSA response, and response in circulating tumor cells. Composite overall response rates were highest in those with BRCA mutations (83.3%) as compared to the other mutations. Patients with ATM mutations had a 36.8% overall response rate, those with CDK12 mutations had a 25% response rate, and patients with PALB2 mutations had a 57.1% composite overall response rate. Secondary endpoints of progression free survival and overall survival were also assessed. Patients with BRCA 1/2 mutations had the longest median progression free survival (PFS) (8.2 months) and median overall survival (OS) (17.7). Those with ATM mutations had a median PFS of 5.6 months and OS 16.6 months. Patients with CDK 12 mutations had worse outcomes with a median PFS of 2.9 months and OS 9.5 months. The patients with PALB2 mutations had a median PFS of 5.3 months and OS 13.9 months. It was therefore noted in this trial that patients with CDK12 mutations seemed to have poorer outcomes with treatment with PARP inhibitors and that while patients with ATM and PALB2 mutations did have some response to treatment, it was less significant than the patients with BRCA 1 and 2 mutations.

The phase III PROfound Trial evaluated olaparib versus second generation hormonal therapy in patients with BRCA mutations as well as in those with 13 other genes implicated in HRR (26). Many of these genes are quite rare and only one or two patients with many of the mutations were included in the trial. The primary endpoint was progression free survival. Patients with BRCA2 mutations were again found to have the best response to treatment with olaparib, with median PFS being 10.84 months versus 3.48 months in the control arm. Patients with RAD51B mutations had similar responses with a median PFS 10.89 months as compared to 1.77 months in the control arm. Patients with RAD54L had a median PFS of 7.2 months in the PROfound Trial. Those with mutations in CDK12, ATM, and CHEK2 all had a median PFS of about 5 months. The U.S. Food and Drug Administration (FDA) approved olaparib for patients who had progression after treatment with enzalutamide or abiraterone with somatic or germline mutations in HRR genes (including BRCA1 and 2, ATM, BARD1, BRIP1, CDK12, CHEK1, CHEK2, FANCL, PALB2, RAD51B, RAD51C, RAD51D, or RAD54L) based on this data (27).

The TRITON-2 study evaluated the use of rucaparib in patients with HRR deficiency. A separate analysis was performed on the data for patients with non-BRCA mutations (28). In patients with ATM mutations, only 2 of 19 patients had radiologic responses and only 2 of 49 of these patients had PSA responses. The patients with CDK12 mutations also had low response rates with 0 of 10 patients having radiologic responses and 1 of 15 having PSA responses. 1 of 9 patients with CHEK2 mutations had a radiologic response and 2 of 12 of these patients had PSA responses. The phase III TRITON3 study plans to compare rucaparib with abiraterone, enzalutamide, and docetaxel in patients with metastatic CRPC and HRR mutations (29). Another study underway at the National Cancer Institute is the CASPAR trial (30). This is also evaluating the combination of Rucaparib with Enzalutamide in patients with mCRPC both with and without HRR mutations (NCT04455750) (30).

Niraparib has also been evaluated in patients with metastatic CRPC and non-BRCA HRR mutations. This was evaluated in the phase II GALAHAD trial (31). The trial included 47 patients with measurable disease and non-BRCA mutations including ATM, FANCA, PALB2, CHEK2, BRIP1, and HDAC2. The patients with non-BRCA mutations were assessed as one cohort. These patients had a lower objective response rate (10.6%) as compared to the patients with BRCA mutations (34.2%) (31).

The MAGNITUDE trail studied Niraparib in combination with abiraterone acetate and prednisone (AAP) vs Niraparib alone (32). This study is still ongoing but has shown benefit of combination therapy in both those with specific BRCA1/BRCA2 mutations and all with HRR mutations. Radiographic PFS is higher in both subgroups, reducing the risk of progression or death by 47% (16.6 vs 10.9 mos in the BRCA1/2 group) (16.5 vs 13.7 mo in the HRR+ group (32).

Talazoparib is another PARP inhibitor which has been evaluated in this population. This was evaluated in the phase II TALAPRO-1 study (33). Objective response rates were again lower in the patients with PALB2 and ATM mutations as compared to patients with BRCA mutations (33).

In review of these studies, it seems that PARP inhibitors result in less dramatic responses in patients with non-BRCA HRR mutations. However, given a lack of treatments with better data, PARP inhibitors remain an important therapeutic option for these patients. Given the relative frequency of non-BRCA HRD alterations, future trials evaluating responsiveness to PARP inhibitors will inform clinical management in this population.

There is an interest in evaluating combinations of PARP inhibitors with other known treatments to improve efficacy. The PROpel trial is a phase 3 trial studying the use of abiraterone with olaparib in those with mCRPC in the first line setting. The genes assessed were ATM, BRCA1, BRCA2, BARD1, BRIP1, CDK12, CHEK1, CHEK2, FACL, PALB2, RAD51B, RAD51C, RAD51D, and RAD54L (34). However, patients were enrolled regardless of HRR mutation status. Patients were classified into three groups: HRR mutated (28.4%), non mutated (69.3%), and unknown (2.3%). Those with HRR mutations had slightly more significant improvement with abiraterone and olaparib. The study found that median image-based PFS was significantly longer in all patients who were treated with abiraterone and olaparib vs. abiraterone alone (24.8 vs. 16.6 months) despite mutation status. Overall survival was immature. The combination of abiraterone and olaparib has since been approved by the U.S. FDA (35). This study confirms the importance of continuing trials studying combination therapy in both those with and without HRR mutations (34).

PLATI-PARP is a phase II trial being conducted at the University of Washington studying the effectiveness of docetaxel with carboplatin followed by rucaparib in the treatment of metastatic CRPC (36). The study will be analyzing patients with BRCA1 and 2 mutations but will also include ATM gene mutations (NCT03442556) (36).

A phase II trial called BRCAAway will randomly assign patients with BRCA 1/2, and ATM mutations to receive either abiraterone, olaparib, or the combination of abiraterone and olaparib (37). The study will also assess treatment of patients with more rare types of DNA repair gene mutations including FANCA, PALB2, RAD51, ERCC3, MRE11, NBN, MLH3, CDK12, CHEK2, HDAC2, ATR, PMS2, GEN1, MSH2, MSH6, BRIP1, or FAM175A with olaparib (NCT03012321) (37). The phase III study TALAPRO-2 is a double-blinded randomized placebo-controlled trial aimed to compare talazoparib with enzalutamide versus enzalutamide monotherapy in mCRPC patients (38).

There is an especially low rate of response to PARP inhibitors in patients with CDK12 mutations (23). CDK4/6 inhibitors are commonly used in the treatment of hormone-receptor positive breast cancer and have shown anti-tumor effects in preclinical studies of CRPC cells (39). It is also thought that loss of function alterations in CDK12 may confer sensitivity to immunotherapy. Based on this data, there is currently an ongoing phase II trial evaluating abemaciclib alone versus abemaciclib plus atezolizumab in patients with CDK12 mutations (39).

### Enhancement of PARP inhibitor activity in HRR mutations

An *in vitro* study by Zhou et al. (2021) took a different approach to enhance the efficacy of PARP inhibitors with CRPC cells (40). Their hypothesis was that the addition of an agent which downregulates the ATM/ATR, and RAD51 pathways would enhance the effectiveness of the PARP inhibitors. Previous research has shown that the proto-oncogene, MET, is highly expressed in CRPC (41). It has been proposed previously that MET may phosphorylate regulatory checkpoints pathways. Zhou et al. examined the growth of prostate cancer cells when MET expression was silenced using crizotinib. Crizotinib is a tyrosine kinase inhibitor for MET and other mutations. It is regularly used to treat other types of cancer such as non-small cell lung cancer (42). The study found a synergistic antineoplastic effect on colony formation in migration assays with combination therapy compared to monotherapy alone. They analyzed the expression level of DNA damage markers in the prostate cancer cell lines. Results of immunofluorescence analysis showed increased DNA damage in those exposed to combination treatment compared to monotherapy. They also found that ATM, ATR, and RAD51 were decreased with combination therapy. Therefore, they concluded that MET silencing downregulated these pathways and enhanced sensitivity to PARP inhibition. This led to the promising idea of combination therapy of PARP and MET inhibition in those with the above mutations.

## Other rare DDR pathway mutations

### MRN complex

The MRN complex is also part of homologous recombination repair. This complex is made up of the MRE11, RAD50, and NBS1 enzymes (43, 44). Instead of recognizing sequences that are damaged and correcting it, the MRN complex can detect DNA breaks and induce apoptosis of these cells instead of inducing repair. MRE11 is specifically activated when irreversible DNA damage occurs and augments apoptosis. A high level of MRE11 expression in prostate cancer tissue has been associated with poor outcomes, including increased risk of recurrence and decreased overall survival (43). There is interest in finding a treatment which impairs the MRN complex in order to accumulate DNA damage in the tumor cell and thus prevent growth. An MRN-ATM pathway inhibitor, called mirin, has been investigated *in vitro* for this purpose. Mirin inhibits MRE11 activity and seems to inhibit androgen-dependent transcription and thus replication of tumor cells in prostate cancer (44). NBS1 appears to be a prostate-cancer susceptibility gene. Mutations in this are more common in familial types of prostate cancer than sporadic. Additionally, NBS1 mutations seem to be more prevalent in patients who have higher grade disease (45).

### Histone deacetylases (HDAC)

Histones are protein scaffolds that allow genomic DNA to be systematically wrapped and packaged. Modifications to these proteins by acetylation or deacetylation control how tightly the DNA is wound around the histones and thus allows for tight control of gene expression. Histone deacetylases (HDACs) have been shown to be overexpressed in prostate cancer (46). Increased expression of HDACs have been associated with enhanced tumor cell proliferation, aggressive phenotype, and worse clinical outcomes such as PSA relapse-free survival. HDACs also have non-histone targets including Ku70, which is a crucial component of the non-homologous end-joining DNA repair pathway (46). Additionally, HDACs play a role in HR DNA damage repair. Thus, HDAC inhibitors are now being investigated in combination with PARP inhibitors as a way to improve antitumor efficacy.

Vornistat is a small molecule inhibitor of class I and II HDACs and has been shown to be effective in inhibiting growth in preclinical prostate cancer cell lines (47). However, In a phase II trial evaluating vorinostat in mCRPC patients, there was only best objective response in 2 patients (7%) and there was significant grade 3 toxicity limiting efficacy assessment (47).

### PARP and HDAC inhibitors

In preclinical studies, a PARP inhibitor (veliparib) and vornistat, a pan-HDAC inhibitor, have been shown to have a synergistic effect leading to decreased expression and stability of the HR DNA repair protein BRCA1 (48). Thus, there is an active phase 1 dose-escalation trial investigating Talazoparib (PARP inhibitor) in combination with Belinostat (HDAC inhibitor) for metastatic cancers including mCRPC (NCT04703920) (49).

## Conclusion

The treatment of metastatic castrate resistant prostate cancer is evolving to focus on a more targeted approach. Identifying treatments for patients with mutations in both HRR and DDR regulation has led to improved progression free survival and overall survival in these patients. Many clinical trials are continuing to target these rare pathways (**Table 1**). With further research, the course of disease for those with CRPC may be able to be significantly improved.

**Table 1:** Clinical trials investigating DDR mutations in metastatic castration-resistant prostate cancer.

Agent / Trial	Trial summary	Outcome	Adverse events (%)	Clinical outcome
PARP Inhibitor				
Olaparib				
TOPARB-B	Open-label, single arm, Phase 2, Olaparib 400 mg or 300 mg twice daily in mCRPC patients with DDR aberrations. (NCT01682772)	Efficacy and safety	Most common grade 3-4 adverse events in both cohorts was anemia (15 patients [31%] in the 300 mg cohort and 18 patients [37%] in the 400 mg cohort.) One death possibly related to treatment (myocardial infarction)	Confirmed composite response was achieved in 25 patients (54·3%; 95% CI 39·0–69·1) in the 400 mg cohort, and 18 patients (39·1%; 25·1–54·6) in the 300 mg cohort. Median radiographic PFS 5.5 months (95% CI 4.4-8.3) in the 400 mg cohort and 5.6 mo (3.7-7.7) in the 300 mg cohort. Median OS 14.3 mo (9.7-14.3) vs 10.1 mo (9.0-17.7).
PROfound	Open-label, Randomized Phase 3, Olaparib Versus Enzalutamide or Abiraterone Acetate in mCRPC patients with HR gene defieincy. (NCT02987543)	Efficacy and safety	Most common adverse events of any grade were anemia, nausea, fatigue or asthenia. 11 cases [4%] in the Olaparib group had pulmonary embolism compared to 1 [1%] in the control arm.	Median progression free survival (7.4 months vs 3.6 month in Olaparib vs control; hazard ratio for progression or death 0.34; 95% confidence interval, 0.25. to 0.47; P<0.001) Median OS 18.5 mo in Olaparib group and 15.1 months in control group.
Rucaparib				
TRITON2	Open-label, Single Group Phase 2, Rucaparib in mCRPC patients with HR gene deficiency. (NCT02952534)	Efficacy and safety	Most frequent grade 3-4 adverse event was anemia (20/115 patients [25.2%])	ORR 43.5% (95% CI, 31.0% to 56.7%) and 50.8% (95% CI, 38.1%-63.4%) by independent radiology review and investigators respectively. Median time to PSA progression 6.5 mo (95% CI, 5.9-7.8). Median rPFS was 9 mo (8.3-13.5). OS not yet matured. Estimated 12 mo OS 73% (62.9-80.7).
TRITON3	Open-label, Randomized Phase 3, Olaparib vs physician’s choice of therapy for mCRPC patients with HR gene deficiency. (NCT02975934)	Efficacy and safety	Active, not recruiting	Active, not recruiting
CASPAR	Double-blind, Randomized Phase 3, Rucaparib in combination with Enzalutamide in mCRPC patients (NCT04455750)	Efficacy and Safety	Recruiting	Recruiting
Niraparib				
GALAHAD	Open-label, Single Group Phase 2, Niraparib in mCRPC patients with DNA-Repair Anomalies (NCT02854436)	Efficacy and safety	Most common grade 3-4 adverse event was hematologic (anemia 95/289 patients [54%]; thrombocytopenia 47 [16%]; neutropenia 28 [10%])	ORR 34.2% (95% CI 23.7-46%) Median rPFS 8.2 mo (95% CI, 5.2-11.1). Median OS 12.6 mo (9.2-15.7) among patients with biallelic BRCA alterations.
MAGNITUDE	Double-blind, Randomized Phase 3, Niraparib in combination with Abiraterone Acetate and Prednisone in mCRPC patients with and without gene alterations (NCT03748641)	Efficacy and safety	Active, not recruiting	Radiographic PFS 16.6 mos in BRCA1/2 subgroup 95% CI 0.36-0.79), 16.5 mos in all HRR+ patients (95% CI 0.56-0.96) OS data is immature
Talazoparib				
TALAPRO-1	Open-label, Single Group Phase 2, Talazoparib in mCRPC with DNA Repair Defects (NCT03148795)	Efficacy and safety	Most common grade 3-4 adverse events was hematologic (anemia 39/127 [31%]; thrombocytopenia 11 [9%], neutropenia 10 [8%])	ORR 29.8% (95% CI 21.2-39.6%) Median rPFS 5.6 mo (3.7-8.8)
TALAPRO-2	Double-blind, randomized Phase 3 Placebo-controlled study of talazoparib with enzalutamide vs enzalutamide monotherapy in mCRPC patients (NCT03395197)	Safety and Efficacy	Active, not recruiting	Active, not recruiting
Combination PARP Inhibitor				
PLATI-PARP	Open-label, Single Group Phase 2, Induction Docetaxel and Carboplatin followed by maintenance Rucaparib in mCRPC patients with HR DNA repair deficiency (NCT03442556)	Efficacy and safety	Recruiting	Recruiting
BRCAAWAY	Open-label, Randomized, Crossover Assignment Phase 2, Abiraterone, Olaparib, or Abiraterone + Olaparib in mCRPC patients with DNA repair defects (NCT03012321)	Efficacy and safety	Recruiting	Recruiting
PROpel	Randomized double-blind, placebo-controlled Phase 3, Olaparib + Abiraterone Relative to Placebo + Abiraterone as first line in mCRPC (NCT03732820)	Efficacy and safety	Most common AEs were anemia [46%], fatigue/asthenia [37.2%], nausea [28.1%]. 15.1% had Grade 3 ≥ anemia.	Investigator assessed image based PFS 24.8 vs 16.6 mo (HR 0.66, 95% CI, 0.54-0.81) ORR 58.4% vs 48.1% (OR 1.60; 95% CI, 1.02-2.53) abiraterone and Olaparib vs abiraterone and placebo arm.
CDK Inhibitor				
Abemaciclib	Open-label, Randomized, Parallel Assignment Phase 2 Abemaciclib with or without atezolizumab in mCRPC patients (NCT04751929)	Efficacy and safety, dose limiting toxicity	Recruiting	Recruiting
HDAC Inhibitor				
Vorinostat	Open-label, Single Group Phase 2 Vorinostat in patients with advanced prostate cancer that have progressed on one prior chemotherapy (NCT00330161)	Efficacy and safety	Most common AEs were fatigue [81%], nausea [74%], anorexia [59%], vomiting [33%], diarrhea [33%], weight loss [26%] 1 patient had grade 4 thrombosis.	Best ORR 7% [2 patients] was stable disease. Median time to progression 2.8 months and median OS was 11.7 months.
Talazoparib with Belinostat	Open-label, Single Group Phase 1 Talazoparib in Combination with Belinostat for Metastatic Breast Cancer, mCRPC, and Metastatic Ovarian Cancer (NCT04703920)	Dose-Escalation	Recruiting	Recruiting

## Author contributions

DM is first author, QS is second author, JC is third author, and MB is senior author. All authors contributed to the article and approved the submitted version.

## References

[1] Siegel R Ma J Zou Z Jemal A. Cancer statistics, 2014. CA Cancer J Clin (2014) 64:9–29. doi: 10.3322/caac.21208 24399786

[2] Ferlay J Steliarova-Foucher E Lortet-Tieulent J Rosso S Coebergh JWW Comber H. Cancer incidence and mortality patterns in Europe: estimates for 40 countries in 2012. Eur J Cancer. (2013) 49:1374–403. doi: 10.1016/j.ejca.2012.12.027 23485231

[3] Rawla P. Epidemiology of prostate cancer. World J Oncol (2019) 10(2): 63–9. doi: 10.14740/wjon1191 PMC6497009 31068988

[4] Zhou Y Bolton EC Jones JO. Androgens and androgen receptor signaling in prostate tumorigenesis. J Mol Endocrinol (2015) 54(1):R15–29. doi: 10.1530/JME-14-0203 25351819

[5] Taylor BS Schultz N Hieronymus H Gopalan A Xiao Y Carver BS. Integrative genomic profiling of human prostate cancer. Cancer Cell (2010) 18(1):P11–22. doi: 10.1016/j.ccr.2010.05.026 PMC3198787 20579941

[6] Kirby M Hirst C Crawford ED. Characterising the castration-resistant prostate cancer population: A systematic review. Int J Clin Pract (2011) 65(11):L1180–92. doi: 10.1111/j.1742-1241.2011.02799.x 21995694

[7] Turco F Gillessen S Cathomas R Buttigliero C Vogl UM. Treatment landscape for patients with castration-resistant prostate cancer: Patient selection and unmet clinical needs. Res Rep Urology. (2022) 14:339–50. doi: 10.2147/RRU.S360444 PMC9529226 36199275

[8] Michels J Montemurro T Murray N Kollmannsberger C Chi KN. First- and second-line chemotherapy with docetaxel or mitoxantrone in patients with hormone-refractory prostate cancer: does sequence matter? Cancer (2006) 106(5):1041–6. doi: 10.1002/cncr.21695 16456811

[9] Oh WK Manola J Babcic V Harnam N Kantoff PW. Response to second-line chemotherapy in patients with hormone refractory prostate cancer receiving two sequences of mitoxantrone and taxanes. Urology (2006) 67(6):1235–40. doi: 10.1016/j.urology.2006.01.006 16765185

[10] Berthold DR Pond GR de Wit R Eisenberger M Tannock IF. Survival and psa response of patients in the TAX 327 study who crossed over to receive docetaxel after mitoxantrone or vice versa. Ann Oncol (2008) 19(10):1749–53. doi: 10.1093/annonc/mdn288 18487550

[11] Higano CS Schellhammer PF Small EJ Burch PA Nemunaitis J Yuh L. Integrated data from 2 randomized, double-blind, placebo-controlled, phase 3 trials of active cellular immunotherapy with sipuleucel-T in advanced prostate cancer. Cancer (2009) 115(16):3670–79. doi: 10.1002/cncr.24429 19536890

[12] Deshayes E Roumiguie M Thibault C Beuzeboc P Cachin F Hennequin C. Radium 223 dichloride for prostate cancer treatment. Drugs Des Devel Ther (2017) 11:2643–51. doi: 10.2147/DDDT.S122417 PMC5593411 28919714

[13] Hennrich U Eder M. [177 Lu] Lu-PSMA-617 (Pluvicto): The first FDA-approved radiotherapeutical for treatment of prostate cancer. Pharm (Basel). (2022) 15(10):1292. doi: 10.3390/ph15101292 PMC9608311 36297404

[14] Wright WD Shah SS Heyer WD. Homologous recombination and the repair of DNA double-strand breaks. J Biol Chem (2018) 293(27):10524–35. doi: 10.1074/jbc.TM118.000372 PMC6036207 29599286

[15] Liang Y Lin S-Y Brunicardi FC Goss J Li K. DNA Damage response pathways in tumor suppression and cancer treatment. World J Surg (2009) 33(4):661–6. doi: 10.1007/s002698-008-9840-1 19034564

[16] Mitra A Fisher C Foster CS Jameson C Barbachanno Y Bartlett J. Prostate cancer in male BRCA1 and BRCA2 mutation carriers has a more aggressive phenotype. Br J Cancer. (2008) 98(2):502–7. doi: 10.1038/sj.bjc.6604132 PMC2361443 18182994

[17] Tryggvadottir L Vidarsdottir L Thorgeirsson T Jonasson JG Olafsdottir EJ Olafsdottir GH. Prostate cancer progression and survival in BRCA2 mutation carriers. J Natl Cancer Inst (2007) 99(12):929–35. doi: 10.1093/jnci/djm005 17565157

[18] Castro E Romero-Laorden N Del Pozo A Lozano R Medina A Puente J. PROREPAIR-b: A prospective cohort study of the impact of germline DNA repair mutations on the outcomes of patients with metastatic castration-resistant prostate cancer. J Clin Oncol (2019) 37(6):490–503. doi: 10.1200/JCO.18.00358 30625039

[19] Thompson D Easton DF. Breast cancer linkage consortium. Cancer incidence BRCA1 Mutat carriers. J Natl Cancer Inst (2002) 94(18):1358–65. doi: 10.1093/jnci/94.18.1358 12237281

[20] Messina C Cattrini C Soldato D Vallome G Caffo O. BRCA mutations in prostate cancer: Prognostic and predictive implications. J Oncol (2020) 2020: 1–7. doi: 10.1155/2020/4986365 PMC7492871 32963528

[21] Wokolorczyk D Kluzniak W Stempa K Rusak B Huzarski T Gronwald J. PALB2 mutations and prostate cancer risk and survival. Br J Cancer. (2021) 125(4):569–75. doi: 10.1038/s41416-021-01410-0 PMC8368211 34006922

[22] Neeb A Herranz N Gallego SA Miranda S Buroni L Yuan W. Advanced prostate cancer with ATM loss: PARP and ATR inhibitors. Eur Urol. (2021) 79(2):200–11. doi: 10.1016/j.eururo.2020.10.029 33176972

[23] Antonarakis ES Velho PI Fu W Wang H Agarwal N Santos VS. CKD12-altered prostate cancer: Clinical features and therapeutic outcomes to standard systemic therapies, poly (ADP-ribose) polymerase inhibitors, and PD-1 inhibitors. JCO Precis Oncol (2020) 4:370–801. doi: 10.1200/po.19.00399 32462107 PMC7252221

[24] Wilkes DC Sailer V Xue H Cheng H Collins CC. A germline FANCA alteration that is associated with increased sensitivity to DNA damaging agents. Cold Springs Harb Mol Case Stud (2017) 3(5):a001487. doi: 10.1101/mcs.a001487 PMC5593159 28864460

[25] Mateo J Porta N Bianchini D McGovern U Elliott T Jones R. Olaparib in patients with metastatic castration-resistant prostate cancer with DNA repair gene aberrations (TOPARP-b): A multicentre, open-label, randomised, phase 2 trial. Lancet Oncol (2020) 21(1):162–74. doi: 10.1016/S1470-2045(19)30684-9 PMC6941219 31806540

[26] De Bono J Mateo J Fizazi K Saad F Shore N Sandhu S. Olaparib for metastatic castration-resistant prostate cancer. N Engl J Med (2020) 382(22):2091–102. doi: 10.1056/NEJMoa1911440 32343890

[27] Targeted Oncology. FDA Grants priority review to olaparib combination for mCRPC. Available at: https://www.targetedonc.com/view/fda-grants-priority-review-to-olaparib-combination-for-mcrpc (Accessed February, 27th, 2023).

[28] Abida W Patnaik A Campbell D Shapiro J Bryce AH McDermott R. Rucaparib in men with metastatic castration-resistant prostate cancer harboring a BRCA1 or BRCA2 gene alteration. J Clin Oncol (2020) 38(32):3763–72. doi: 10.1200/JCO.20.01035 PMC7655021 32795228

[29] Clovis Oncology, Inc. A study of rucaparib versus physician’s choice of therapy in patients with metastatic castration-resistant prostate cancer and homologous recombination gene deficiency (TRITON3). Boulder, Co :Clovis Oncology, Inc. (2022).

[30] National Cancer Institute. A clinical study evaluating the benefit of adding rucaparib to enzalutamide for men with metastatic prostate cancer that has become resistant to testosterone-deprivation therapy (CASPAR). Chicago, IL: Alliance for Clinical Trials in Oncology. (2022).

[31] Smith MR Scher HI Sandhu S Efstathiou E Lara PN Yu EY. Niraparib in patients with metastatic castration-resistant prostate cancer and DNA repair gene defects (GALAHAD): A multicentre, open-label, phase 2 trial. Lancet Oncol (2022) 23(2):362–73. doi: 10.1016/S1470-2045(21)00757-9 PMC9361481 35131040

[32] Chi KN Rathkopf DE Smith MR Efstathiou E Attard G Olmos D. (2022). Phase 3 MAGNITUDE study: First results of niraparib (NIRA) with abiraterone acetate and prednisone (AAP) as first-line therapy in patients (pts) with metastatic castration-resistant prostate cancer (mCRPC) with and without homologous recombination repair (HRR) gene alterations, in: ASCO GU Symposium, 40(6). doi: 10.1200/JCO.2022.40.6_suppl.012

[33] De Bono JS Mehra N Scagliotti GV Castro E Dorff T Stirling A. Talazoparib monotherapy in metastatic castration-resistant prostate cancer with DNA repair alterations (TALAPRO-1): an open label, phase II trial. Lancet Oncol (2021) 22(9):1250–64. doi: 10.1016/S1470-2045(21)00376-4 34388386

[34] Clarke NW Ch M Armstrong AJ Thiery-Vuillemin A Oya M Shore N. Abiraterone and olaparib for metastatic castration-resistant prostate cancer. N Engl J Med (2022) 1(9):2091–102. doi: 10.1056/EVIDDoa2200043

[35] AstraZeneca. Update on US regulatory priority review of lynparza in combination with abiraterone in metastatic castration-resistant prostate cancer. Available at: https://www.astrazeneca.com/media-centre/press-releases/2022/update-on-us-review-of-lynparza-propel-snda.html (Accessed February 27th, 2023).

[36] University of Washington. Docetaxel, carboplatin, and rucaparib camsylate in treating patients with metastatic castration resistant prostate cancer with homologous recombination DNA repair deficiency. Seattle, WA: University of Washington. (2022).

[37] Northwestern University. Abiraterone/Prednisone, olaparib, or Abiraterone/Prednisone + olaparib in patients with metastatic castration-resistant prostate cancer with DNA repair defects. Chicago, IL. (2022).

[38] Agarwal N Azad A Shore ND Carles N Fay AP Dunshee C. Talazoparib plus enzalutamide in metastatic castration-resistant prostate cancer: TALAPRO-2 phase III study design. Future Oncol (2022) 18(4):425–36. doi: 10.2217/fon-2021-0811 35080190

[39] Rao R Kwak L Reimers MA Reichert ZR Thyagarajan B. A phase II trial of abemaciclib (abema) and atezolizumab (atezo) in unselected and CDK12-loss metastatic castration-resistant prostate cancer (mCRPC). JCO Precis Oncol (2022) 40(6). doi: 10.1200/JCO.2022.40.6_suppl.TPS213

[40] Zhou S Dai Z Wang L Gao X Yang L Wang Z. MET inhibition enhances PARP inhibitor efficacy in castration-resistant prostate cancer by suppressing the ATM/ATR and PI3K/AKT pathways. J Cell Mol Med (2021) 25(24):11157–69. doi: 10.1111/jcmm.17037 PMC8650038 34761497

[41] Pisters LL Troncoso P Zhau HE Li W von Eschenbach AC Chung LW. C-met proto-oncogene expression in benign and malignant human prostate tissues. J Urol. (1995) 154(1):293–8. doi: 10.1016/S0022-5347(01)67297-5 7539865

[42] Ayoub NM Al-Shami KM Alqudah MA Mhaidat NM. Crizotinib, a MET inhibitor, inhibits growth, migration, and invasion of breast cancer cells *in vitro* and synergizes with chemotherapeutic agents. Onco Targets Ther (2017) 10:4869–83. doi: 10.2147/OTT.S148604 PMC5634371 29042798

[43] Wang J Xu WH Yu W Zhu Y Qin XJ Zhang HL. Elevated MRE11 expression associated with progression and poor outcome in prostate cancer. J Cancer. (2019) 10(18):4333–40. doi: 10.7150/jca.31454 PMC6691708 31413753

[44] Bian L Meng Y Zhang M Li D. MRE11-RAD50-NBS1 complex alteration and DNA damage response: Implications for cancer treatment. Mol Cancer. (2019) 18(1):169. doi: 10.1186/s12943-019-1100-5 31767017 PMC6878665

[45] Zhen JT Syed J Nguyen KA Leapman MS Agarwal N. Genetic testing for hereditary prostate cancer: Current status and limitations. Cancer (2018) 124(15):3105–17. doi: 10.1002/cncr.31316 29669169

[46] Abbas A Gupta S. The role of histone deacetylases in prostate cancer. Epigenetics (2008) 3(6):300–9. doi: 10.4161/epi.3.6.7273 PMC2683066 19029799

[47] Bradley D Rathkopf D Dunn R Stadler WM Liu G. Vorinostat in advanced prostate cancer patients progressing on prior chemotherapy (National cancer institute trial 6862): Trial results and interleukin-6 analysis: a study by the department of defense prostate cancer clinical trial consortium and university of Chicago phase 2 consortium. Cancer (2009) 115(23):5541–9. doi: 10.1002/cncr.24597 PMC2917101 19711464

[48] Yin L Liu Y Peng Y Peng Y Yu X Gao Y. PARP inhibitor veliparib and HDAC inhibitor SAHA synergistically co-target the UHRF1/BRCA1 DNA damage repair complex in prostate cancer cells. J Exp Clin Cancer Res (2018) 37:153. doi: 10.1186/s13046-018-0810-7 30012171 PMC6048811

[49] University of Michigan Rogel Cancer Center. Talazoparib in combination with belinostat for metastatic breast cancer, metastatic castration resistant prostate cancer, and metastatic ovarian cancer. Ann Arbor, MI: University of Michigan Rogel Cancer Center. (2022).

